# MicroRNA miR-874-3p inhibits osteoporosis by targeting leptin (LEP)

**DOI:** 10.1080/21655979.2021.2009618

**Published:** 2021-12-11

**Authors:** Ling Mei, Min Li, Tao Zhang

**Affiliations:** a Department of Orthopedic, Wuhan Hospital of Traditional Chinese Medicine, Wuhan, Hubei, China; b Department of Cardiovascular, Wuhan Hospital of Traditional Chinese Medicine, Wuhan, Hubei, China; c The First Clinical Medical College, Hubei University of Chinese Medicines, Wuhan, Hubei, China

**Keywords:** Osteoblast differentiation, hBMSCs, miR-874-3p, LEP, osteoporosis

## Abstract

MicroRNAs (miRNAs) regulate osteogenic differentiation and influence osteoporosis (OP). The aim of this study was to determine the potential role of miR-874-3p in OP. The expression levels of miR-874-3p and leptin (LEP) in the femoral neck trabeculae of 35 patients with or without OP were measured by quantitative reverse transcription-polymerase chain reaction (qRT-PCR). The effects of miR-874-3p or LEP on the cell proliferation and alkaline phosphatase (ALP), runt-related transcription factor 2 (RUNX2), osteocalcin (OCN), and osterix (OSX) levels were observed by upregulating miR-874-3p in human bone marrow mesenchymal stem cells (hBMSCs). Additionally, calcium deposition levels were evaluated using alizarin red staining (ARS). Molecular mechanisms of miR-874-3p and LEP underlying the osteogenic differentiation of hBMSCs were also evaluated using bioinformatics analysis, luciferase reporter assays, and RNA pull-down assays. The miR-874-3p levels were significantly lower in the femoral neck trabeculae of patients with OP than those of the control group, while the opposite was observed regarding the levels of LEP. Expression levels of miR-874-3p in hBMSCs were upregulated during osteogenic differentiation, while those of LEP were downregulated. Moreover, miR-874-3p upregulation promoted ALP, RUNX2, OCN, and OSX mRNA expression, cell proliferation, and calcium deposition in hBMSCs. LEP was found to be a target gene of miR-874-3p. Overexpression of LEP inhibited the expression of osteoblast markers and reversed the effect of osteogenic differentiation induced by the upregulation of miR-874-3p. In conclusion, miR-874-3p promoted the proliferation and differentiation of hBMSCs by downregulating the expression of LEP, thus inhibiting OP.

**Abbreviations :** miRNAs: microRNAs; OP: osteoporosis; hBMSCs: human Bone Marrow Mesenchymal stem cells; LEP: leptin; DEGs: differentially expressed genes

## Introduction

Osteoporosis (OP) is a disease characterized by reduced bone density and mass, increased fragility, and susceptibility to fracture [1]. The main pathogenesis of OP is bone metabolic disorder, osteoblast inactivation leading to reduced bone tissue, and facilitated bone resorption [2]. Human bone marrow mesenchymal stem cells (hBMSCs) are pluripotent stem cells located in the mesoderm that can differentiate into chondrocytes, osteoblasts, adipocytes, myocytes, and other types of cells under certain conditions. They have become ideal seed cells for the treatment of bone diseases, and play a crucial role in bone reconstruction and repair in patients with OP [3]. The critical factor for bone regeneration is to improve the osteogenic differentiation of BMSCs; therefore, BMSCs are important for the investigation of OP [4].

MicroRNAs (miRNAs) are involved in the physiological processes of proliferation, apoptosis, migration, and osteogenic differentiation of different cell types by binding to the 3ʹ-untranslated regions (UTRs) of target genes via base pairing [5,6]. It has been reported that the loss of miRNA-mediated mechanisms is a crucial pathological factor in OP and other bone-related diseases [7]. Previous studies have shown that knockout of miR-339 facilitates osteogenic differentiation by targeting distal-less homeobox 5 (DLX5), thereby alleviating OP [8]. miR-210 acts as a key factor in the improvement of OP by promoting the expression of vascular endothelial growth factor (VEGF) and osteoblast differentiation [9]. miR-338-3p can regulate osteogenic differentiation by combining with the 3ʹ-UTRs of the fibroblast growth factor receptor 2 (FGFR2) and runt-related transcription factor 2 (RUNX2) [10]. miR-874-3p is a special type of miRNA that is involved in the development of many cancers [11]. Lin et al. [12] revealed that miR-874 promotes osteoblast differentiation and proliferation in osteoporotic rats. These results indicate that miR-874 plays an active role in bone formation; however, the underlying mechanism by which it regulates bone formation requires further elucidation.

Leptin (LEP) is a 16 kDa peptide hormone that has several functions. It is one of the long-chain helical cytokine family members that is primarily produced by fat cells and is proportional to the size of the fat stored [13]. LEP affects bone remodeling by reducing the viability of osteoclasts, and thus, participates in the pathogenesis of OP [14]. Research by Ye et al. [15] suggests that LEP may be related to an enhanced risk of OP by increasing the expression levels of proinflammatory cytokines. Additionally, studies have shown that miRNAs, such as miR-29a, can regulate bone anabolism by controlling LEP signals [16]. However, it is unclear whether miR-874-3p/LEP is involved in OP.

The purpose of this study was to confirm the abnormal expression of miR-874-3p and LEP in OP and their role in the osteogenic differentiation of hBMSCs. We hypothesized that miR-874-3p may promote osteogenic differentiation of hBMSCs by targeting LEP, and thus may be an effective marker for the prognosis and diagnosis of OP.

## Materials and methods

### Bioinformatics analysis

GSE37558 from Gene Expression Omnibus (GEO) DataSets is an mRNA expression profile that stores the expression levels of genes in OP and control samples. It was used to screen the differentially expressed genes (DEGs) in OP samples with adjusted P < 0.05, log|FC| ≥ 2. The DEGs were then uploaded to Metascape for Gene Ontology (GO) enrichment and key network analysis. After identifying the genes involved in the key pathways and networks, TargetScan Human 7.2 algorithm was used to predict the potential target miRNAs for the identified key genes.

### Organizational collection

Thirty five cases of postmenopausal osteoporotic fractures and non-osteoporotic fractures each were selected for hip replacement in our hospital. The femoral neck bone fragments were cut into small pieces and stored at – 80°C after washing with phosphate-buffered saline (PBS) thrice. None of the patients had any other history of the disease. This study obtained consent from all patients and was approved by the Ethics Committee of our hospital (approval number: 2019107).

### Cell culture and the protocol for osteogenic-differentiation

hBMSCs were obtained from Cyagen (Guangzhou, China) and cultured in α-minimum essential medium (αMEM; Invitrogen, CA, USA) containing 10% fetal bovine serum (FBS; Gibco, NY, USA), 2 mM L-glutamine (Sigma-Aldrich, MO, USA), and 1% penicillin and streptomycin (Gibco). For osteogenic differentiation, hBMSCs were induced for 16 d using fresh osteogenic medium containing 0.1 mM dexamethasone, β-glycerophosphate (10 mM), and ascorbic acid (0.1 mM) (Sigma-Aldrich). The cells were maintained in a cell incubator containing 5% carbon dioxide (CO_2_) at 37°C [17].

### Cell transfection

The miR-874-3p mimic and negative control miR-874-3p mimic (mimic-NC) were purchased from GenePharma (Shanghai, China). hBMSCs were transfected with 100 pmol of the miR-874-3p mimic or mimic-NC using Lipofectamine 2000 transfection reagent (Invitrogen). The overexpressed-LEP (oe-LEP) was synthesized by Nanjing Kaiji Biotech (Nanjing, China). A final concentration of 200 nM oe-LEP was transfected into hBMSCs, while cells in the control group were not transfected.

### Cell proliferation assay

The Cell Counting kit-8 (CCK-8) assay was used to detect the proliferation of hBMSCs after transfection for 48 h. CCK-8 reagent (10 μL; Sigma-Aldrich) was added to each well at 24, 48, and 72 h and incubated at 37°C for 2 h. A microplate reader (Bio-Rad, Hercules, CA, USA) was used to determine the absorbance at 450 nm [18].

### Quantitative real-time polymerase chain reaction (qRT-PCR)

miRNA was extracted using the NucleoSpin® miRNA kit (Macherey Nagel, France). Then, 50 ng of RNA was reverse transcribed using M-MLV reverse transcriptase (Invitrogen), and the cDNA was diluted 1:10 in the PCR reaction. miR-874-3p reactions were performed using miRNA-specific loop primers and amplified by PCR using Taqman miRNAs (Applied Biosystems, CA, USA). Small nuclear RNA (snRNA) U6 (RNU6B Taqman Mirna Assay; Applied Biosystems) was used as a standardized reference marker. These values are represented by 2^−CT^, where C_T_ = C_Tsample_-C_Tcalibrator_. C_T_ is the threshold period difference between the amplitudes of miR-874-3p and snRNA U6, and C_T_ was performed using an ABI PRISM 7700 Sequence Detector and SYBR Green reagents (Applied Biosystems).

mRNA was extracted from cultured cells using the Eastep® Super RNA Extract reagent Kit (Promega, WI, USA), according to the manufacturer’s instructions. cDNA was synthesized from 0.5 μg RNA by Applied Biosystems. Power SYBR Green PCR Master Mix (Promega) was used to perform real-time PCR on Biosystems of Quantstudio 6 flex (Applied Biosystems). Identification of LEP expression was done by C_T_ normalization of glyceraldehyde‐3‐phosphate dehydrogenase (GAPDH) [19]. The primers for miR-874-3p, LEP, U6, and GAPDH are listed in Table 1.

**Table 1.** The sequence of PCR primers in this study

Primer	Sequences
LEP	Forward: 5′-CACACGCAGTCAGTCTCCTC-3′
	Reverse: 5′-CGGAGGTTCTCCAGGTCAT-3′
ALP	Forward: 5′-ATCTTTGGTCTGGCTCCCATG-3′
	Reverse: 5′- TTTCCCGTTCACCGTCCAC-3′
RUNX2	Forward: 5′-GTAGATGGACCTCGGGAACC-3′
	Reverse: 5′-GAGGCGGTCAGAGAACAAAC-3′
OCN	Forward: 5′-GCAATAAGGTAGTGAACAGACTCC-3′
	Reverse: 5′-GTTTGTAGGCGGTCTTCAAGC-3′
OSX	Forward: 5′-ATGGGCTCCTTTCACCTG-3′
	Reverse: 5′-GGGAAAAGGGAGGGTAATC-3′
miR-874-3p	Forward: 5ʹ-GAACTCCACTGTAGCAGAGATGGT-3’
	Reverse: 5ʹ-CATTTTTTCCACTCCTCTTCTCTC-3’
GAPDH	Forward: 5ʹ-TCCTCTGACTTCAACAGCGA-3’
	Reverse: 5ʹ-GGGTCTTACTCCTTGGAGGC-3’
U6	Forward: 5ʹ-CTCGCTTCGGCAGCACA-3’
	Reverse: 5ʹ-AACGCTTCACGAATTTGCGT-3’

### Alizarin red staining (ARS)

As previously described [20], mineral deposition of hBMSCs was observed by staining after osteogenic induction. hBMSCs were maintained in the induction medium for 16 d, then removed and immobilized with 4% paraformaldehyde. Next, the cells were dyed with 1% ARS solution at 25°C. Finally, the culture plates were photographed.

### Dual-luciferase reporter assay

This assay was performed based on a previous study [21]. LEP 3ʹ-UTR fragment was inserted into the pGL3 plasmid to construct the wild-type LEP reporter plasmid. Based on the binding sites of miR-874-3p on LEP 3ʹ-UTR, mutant LEP was constructed using the GeneTailor Site-Directed Mutagenesis System kit (Invitrogen). miR-874-3p mimic or mimic-NC and wild-type LEP plasmid or LEP mutant plasmid were co-transfected into hBMSCs and incubated for 24 h. After transfection, luciferase reporter system (Thermo Fisher Scientific, MA, US) was used to detect the luciferase activity.

### RNA pull-down assay

This assay was carried out in accordance with a previously described method [22]. Briefly, biotinylated miR-874-3p (Bio-miR-874-3p) and Bio-NC were obtained from Genepharma and co-cultured with Dynabeads M-280 Streptavidin (Invitrogen) at 4°C. Beads coated with different probes were incubated in the lysis buffer. RNA extracted was then eluted and purified for qRT-PCR.

### Western blotting

hBMSCs were extracted using the radioimmunoprecipitation assay (RIPA) buffer (Beyotime, China). Proteins (20 µg) were separated by 10% sodium dodecyl sulfate polyacrylamide gel electrophoresis, transferred to a nitrocellulose membrane, washed with TBS containing 0.05% Tween 20 (TBST), and sealed with 5% skim milk at 25°C for 1 h. Next, anti-LEP (1:1000; Abcam, Cambridge, UK) and anti-GAPDH (1:3000; Abcam) antibodies were cultured overnight at 4°C. The membrane was heated at 25°C and incubated with a secondary antibody (1:5000; Proteintech, IL, USA) for 2 h. Images were obtained using enhanced chemiluminescence. The results were analyzed using a Gel-Pro Analyzer (United States Biochemical, OH, USA) [23].

### Statistical analysis

Statistical analysis was performed using the GraphPad Prism 6 software (GraphPad Software, CA, USA). All data are presented as the average mean ± standard deviation (SD). Student’s t-test was used to evaluate the statistical significance of the differences between the two groups. The statistical significance of the differences among multiple groups was analyzed using one-way or two-way analysis of variance (ANOVA) followed by Dunnett’s or Tukey’s multiple comparison test. The relationship between LEP mRNA and miR-874-3p in osteoporotic tissues was evaluated using Pearson analysis. Statistical significance was set at P < 0.05.

## Results

Here, we hypothesized that miR-874-3p promotes osteogenic differentiation of hBMSCs by inhibiting LEP to alleviate OP. First, the genes to be studied were identified as miR-874-3p and LEP via bioinformatics analysis. Moreover, miR-874-3p and LEP were found to be abnormally expressed in OP and in the osteogenic differentiation of hBMSCs. In addition, we evaluated the effects of miR-874-3p and LEP on the proliferation of hBMSCs, calcium deposition, and ALP, RUNX2, OCN, and OSX expression levels. Moreover, we validated the target binding between miR-874-3p and LEP. The results suggest that targeting the miR-874-3p/LEP axis may be a new therapeutic strategy for OP.

### The identification of LEP and miR-874-3p in OP

**Figure 1.**
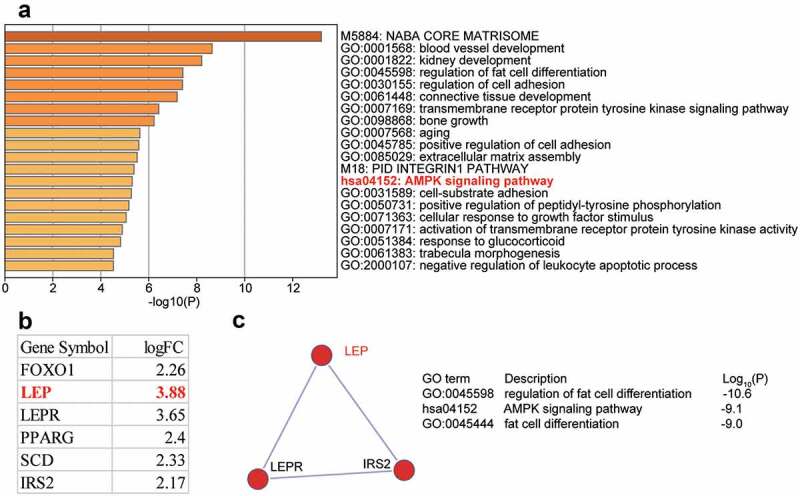
The identification of genes of interest in osteoporosis. (a). The top 20 enriched GO terms of the differentially expressed genes (DEGs) from GSE37558 data series. DEGs selection criteria: adjusted P < 0.05, log|FC|≥2. FC: fold change. (b). The six AMPK signaling pathway-related DEGs and their expression levels in GSE37558 data series. (c). The key network that involves LEP, LEPR, and IRS2 from AMPK signaling pathway

DEGs (n = 88) in the OP and control (GSE37558 [24]) samples were screened using the criteria of adjusted P < 0.05, and log|FC| ≥ 2. Eighty eight genes were uploaded to Meatscape.org for enrichment analysis. AMP-activated protein kinase (AMPK) signaling was observed among the enriched terms. AMPK signaling has been shown to play a crucial role in OP [25–28]. Six DEGs, forkhead box O1 (FOXO1), LEP, leptin receptor (LEPR), peroxisome proliferator-activated receptor gamma (PPARG), stearoyl-CoA desaturase (SCD), and insulin receptor substrate 2 (IRS2), were found to be involved in AMPK signaling (Figure 1(a)). We also investigated GSE37558 expression profile and found that LEP was the most significantly upregulated DEG (Figure 1(b)). Three of the six DEGs, LEP, LEPR, and IRS2, were found in a key network (Figure 1(c)). Due to the limited number of studies reporting the role of LEP in OP, we chose LEP as our gene of interest. As shown in Supplementary Table 1, we used TargetScan Human 7.2 algorithm to predict the potential target miRNAs that target the LEP mRNA, and found that among the top five most ranked miRNAs, miR-874-3p was once reported to promote osteoblast proliferation [12]. Nonetheless, the effect of miR-874-3p on OP by targeting LEP had not yet been studied. Therefore, we selected miR-874-3p as the miRNA of interest in this study.

### miR-874-3p promotes the differentiation of hBMSCs into osteoblasts for the treatment of OP

**Figure 2.**
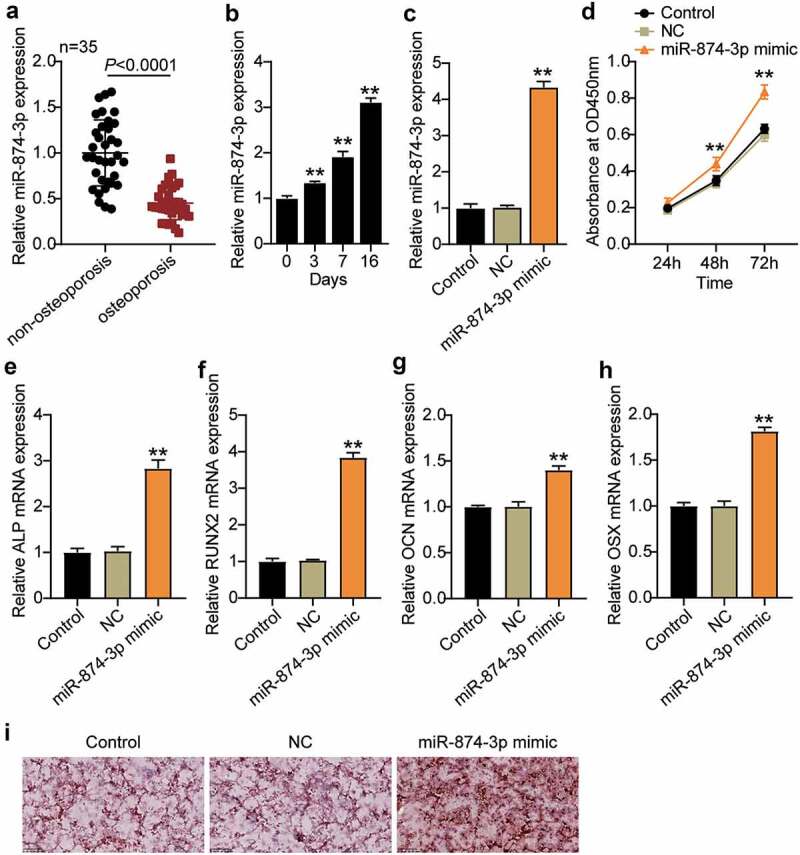
MiR-874-3p promotes the differentiation of hBMSCs into osteoblasts for the treatment of osteoporosis. (a). Determination of the expression of miR-874-3p in femoral neck trabecular bones of OP patients (n = 35) and non-OP patients (n = 35) by qRT-PCR. (b). The expression levels of miR-874-3p were quantified by qRT-PCR on days 0, 3, 7, and 16 after the osteogenic differentiation of hBMSCs. (c). The expression of miR-874-3p was determined by qRT-PCR in hBMSCs transfected with the miR-874-3p mimic or negative control (NC). (d). After 24, 48 and 72 h cultured, cell proliferation was detected by CCK-8 assay in hBMSCs transfected with miR-874-3p mimic. E-H. The expression levels of ALP, RUNX2, OCN and OSX were determined by qRT-PCR in hBMSCs transfected with the miR-874-3p mimic. I. The hBMSCs were overexpressed with miR-874-3p after 16 days of osteogenic differentiation and underwent ARS staining to detect calcium deposition. n = 3, repetition = 3. * P < 0.05, ** P < 0.001. Student’s t-test, One-way or two-way ANOVA were used in statistical analysis. Control, blank control

miR-874-3p levels in OP were measured by qRT-PCR. The results revealed that the miR-874-3p levels in the femoral neck trabecular bones of OP decreased by approximately 50% compared with those in non-OP patients (Figure 2(a)). In addition, osteogenic differentiation of hBMSCs was induced and showed that miR-874-3p expression increased with the induction time (Figure 2(b)). This suggests that miR-874-3p may have beneficial effects in OP.

To investigate the effect of miR-874-3p on the osteogenic differentiation of hBMSCs, the miR-874-3p mimic was transfected into hBMSCs. We found that miR-874-3p expression was successfully increased by approximately four times (Figure 2(c)). Cell proliferation was detected by CCK-8 assay, and the data showed that the absorbance was increased by approximately 1.4 times (Figure 2(d)). Multiple signaling pathways and factors are involved in osteogenic differentiation, of which RUNX2 and OSX are important osteogenic transcription factors that lead to eventual osteoblast differentiation, characterized by calcification of the extracellular matrix [29]. In addition, ALP and OCN also participate in the bone mineralization process [30]. The ALP, RUNX2, OCN, and OSX mRNA levels in hBMSCs transfected with miR-874-3p mimic were found to be elevated by qRT-PCR (Figure 2(e-h)). ARS staining further demonstrated an increased level of calcium nodules in hBMSCs overexpressing miR-874-3p (Figure 2(i)). These results suggest that overexpression of miR-874-3p can promote the differentiation of hBMSCs into osteoblasts, which is feasible for the treatment of OP.

### miR-874-3p negatively regulates the expression of LEP in hBMSCs

**Figure 3.**
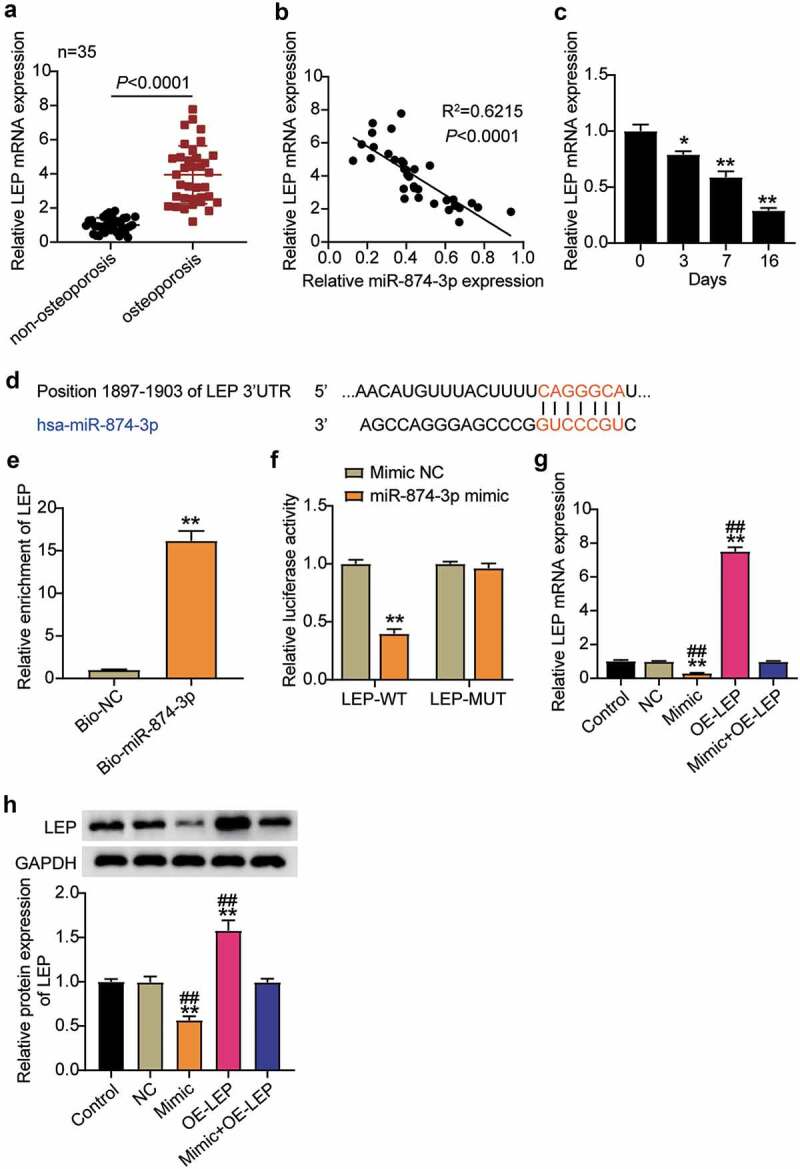
MiR-874-3p negatively regulates the expression of LEP in hBMSCs. (a). Determination of the expression of LEP mRNA in femoral neck trabecular bones of OP patients (n = 35) and non-OP patients (n = 35) by qRT-PCR. (b). Pearson analysis of correlation between miR-874-3p and LEP in femoral neck trabecular bones of OP patients. (c). The expression levels of miR-874-3p were quantified by qRT-PCR on days 0, 3, 7, and 16 during hBMSCs osteogenic differentiation. (d). Schematic diagram of predicted binding sites of LEP in 3ʹ-UTR of miR-874-3p. (e). Detection of LEP enrichment on miR-874-3p in BMSCs by RNA pull-down. (f). Determination of the dual-luciferase activity of hBMSCs transfected with LEP-MUT or LEP-WT and miR-874-3p mimic or mimic NC. (g). Determination of the mRNA expression levels of LEP in hBMSCs transfected with miR-874-3p mimic or/and oe-LEP by qRT-PCR. (h). Determination of the protein expression of LEP in BMSCs transfected with miR-874-3p mimic or/and oe-LEP by Western blot. n = 3, repetition = 3. * P < 0.05, ** P < 0.001 compared with NC group; ## P < 0.001 compared with mimic+oe-LEP group. One-way ANOVA was used in statistical analysis. Control, blank control

The expression levels of LEP in OP and non-OP patients were examined, and the results showed that LEP was upregulated about 4 times in patients with OP compared to non-OP patients (Figure 3(a)). Therefore, the relationship between LEP and miR-874-3p in OP was analyzed and they were found to be negatively correlated (Figure 3(b)). Furthermore, we detected the expression of LEP mRNA in hBMSCs during osteogenic differentiation, and the results indicated that the LEP mRNA expression levels decreased with the extension of induction time, which was contrary to the trend observed in the miR-874-3p expression levels (Figure 3(c)). It was hypothesized that miR-874-3p may negatively regulate the expression of LEP. Then, the binding site between miR-874-3p and LEP was identified using TargetScan (Figure 3(d)). The binding of miR-874-3p to LEP was further evaluated by RNA pull-down assay, which showed that Bio-miR-874-3p increased LEP enrichment levels by approximately 15-fold (Figure 3(e)). Mutant LEP-3ʹ-UTR was obtained by mutating the binding site of LEP, and a dual-luciferase reporter assay was performed. The data indicated that the luciferase activity was downregulated to about 0.5 after the wild-type LEP was co-transfected with miR-874-3p mimic, while the luciferase activity was not affected after the mutant LEP was co-transfected with miR-874-3p mimic (Figure 3(f)). These results indicated that miR-874-3p targets negatively regulated LEP levels. To further verify this, hBMSCs were transfected with miR-874-3p mimic or oe-LEP and it was found that the levels of LEP decreased after upregulation of miR-874-3p, while oe-LEP partially eliminated the influence of miR-874-3p mimic on the expression of LEP (Figure 3(g-h)).

### Overexpression of LEP reverses the effect of miR-874-3p mimic on osteogenic differentiation of hBMSCs

**Figure 4.**
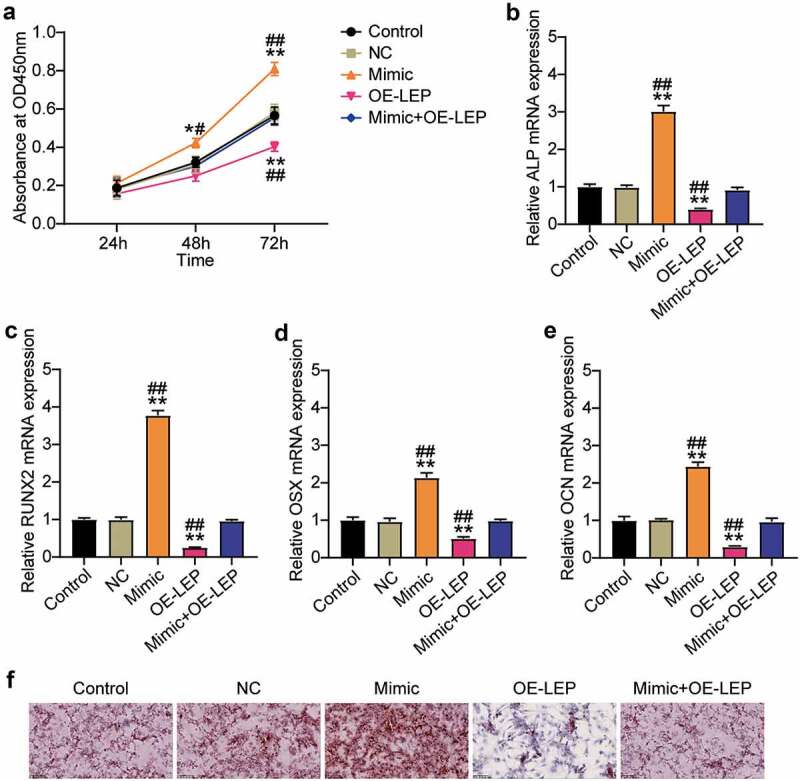
Overexpression of LEP reverses the effect of miR-874-3p mimic on osteogenic differentiation of hBMSCs. (a). After 24, 48 and 72 h culture, cell proliferation was detected by CCK-8 assay in hBMSCs transfected with miR-874-3p mimic or/and oe-LEP. (b)-(e). In the hBMSCs transfected with miR-874-3p mimic or/and oe-LEP, the expression levels of ALP, RUNX2, OCN and OSX were determined by qRT-PCR. (f). The hBMSCs were overexpressed with miR-874-3p mimic or/and LEP after 16 days of osteogenic differentiation and underwent ARS staining to detect calcium deposition. n = 3, repetition = 3. * P < 0.05, ** P < 0.001 compared with NC group; # P < 0.005, ## P < 0.001 compared with mimic+oe-LEP group. One-way or two-way ANOVA was used in statistical analysis. Control, blank control

Since LEP is the target gene of miR-874-3p, we further examined the action mechanisms of LEP and miR-874-3p in the osteogenic differentiation of hBMSCs. Cell proliferation and ALP, RUNX2, OCN, and OSX mRNA levels were detected by the CCK-8 assay and qRT-PCR, respectively. As shown in Figure 4(a), compared with the NC group, overexpression of LEP inhibited the absorbance of hBMSCs by approximately 60% and partially restored the effect of miR-874-3p mimic on hBMSC proliferation. Similarly, upregulation of LEP restrained the mRNA expression levels of ALP, RUNX2, OCN, and OSX to 40, 20, 60, and 30%, respectively, in the NC group, and partially eliminated the miR-874-3p mimic effect (Figure 4(b-e)). ARS staining showed that overexpression of LEP inhibited the formation of mineralized nodules in hBMSCs and counteracted the effect of the miR-874-3p mimic (Figure 4(f)). In conclusion, these results suggest that, contrary to the effect of miR-874-3p, LEP suppressed the osteogenic differentiation of hBMSCs.

## Discussion

Several studies have found that brittle fractures caused by loss of bone strength are the main manifestation of OP [31]. hBMSCs promote osteogenic differentiation and may be involved in callus formation [32,33]. However, the mechanism underlying osteogenic differentiation remains largely unknown. Therefore, a better understanding of osteogenesis of hBMSCs is necessary for the clinical application of OP. In this study, hBMSCs were used as a cell model to investigate the mechanism of miR-874-3p in osteoblast differentiation in OP. Our findings proved that miR-874-3p inhibited OP by targeting LEP.

miR-874-3p regulates cell behaviors, such as cell survival, cell cycle, and apoptosis, and acts as an active factor in bone formation and regulation of OP [11,12,34]. We first examined the expression levels of miR-874-3p in OP and hBMSCs during osteogenic differentiation. Similar to Lin et al. [12], this study revealed that miR-874-3p had a low expression in OP, while its expression levels were elevated in hBMSCs during osteogenic differentiation. Additionally, Kushwaha et al. [35] discovered that miR-874-3p overexpression facilitates osteoblast differentiation and mineralization and promotes bone synthesis by decreasing the levels of histone deacetylase 1. Other studies have shown that miR-874-3p promotes bone formation by targeting VEGFA, a key gene for bone differentiation, which promotes the expression of osteogenic markers, ARS, and ALP activity [36]. Based on these studies, the effect of miR-874-3p on osteogenic differentiation of hBMSCs was further examined, and it was found that miR-874-3p increased the expression levels of bone formation-related genes, such as ALP, RUNX2, OSN, and OSX, and promoted the formation of calcium nodules. This study also found that the proliferation of hBMSCs was upregulated after miR-874-3p overexpression. These results are consistent with those of previous studies, which proved that miR-874-3p is a promoter of osteogenic differentiation and plays a role in the treatment of OP to a certain extent.

Nevertheless, the downstream mechanism by which miR-874-3p plays a role in OP requires further clarification. LEP has been reported to be involved in the formation of OP by decreasing the rate of bone formation and enhancing bone resorption to inhibit the growth of the femur and reduce bone mass, resulting in a reduction in serum insulin-like growth factor (IGF)-I levels, abdominal fat mass, and body weight, while also having a negative effect on OP [37]. Sato et al. [38] found a negative correlation between bone mineral density and serum LEP concentration in adult males. In addition, Hipair et al. [39] studied the relationship between LEP and bone turnover in postmenopausal women with OP and found that LEP in blood circulation may lead to bone loss and is significantly correlated with serum bone markers of high turnover. These reports suggest that LEP plays an important role in osteoblast differentiation in OP. Therefore, we examined the effect of LEP on OP in this study and found that LEP was highly expressed in OP, which is consistent with previous research results. In addition, we found that upregulation of LEP inhibited the proliferation of hBMSCs. This result is similar to that of Kim et al. [40], who reported that very high LEP levels induced the apoptosis of hBMSCs. Furthermore, this study also found that overexpression of LEP inhibited the osteogenic differentiation of hBMSCs by inhibiting the levels of bone formation-related genes, ALP, RUNX2, OSN, and OSX, and inducing calcium nodule formation, thereby indicating that LEP may play a role in worsening OP. Therefore, we identified LEP as a potential target gene for miR-874-3p by bioinformatics analysis, which was subsequently verified to be a direct target by RNA pull-down and luciferase assays. Knowing the effect of miR-874-3p on osteogenic differentiation, the expression levels of osteogenesis-related genes and calcium nodule formation in hBMSCs may be mediated by the downregulation of LEP.

LEP originates from bone marrow adipocytes, chondrocytes, and osteoblasts, and has a direct anabolic effect on osteoblasts and chondrocytes [41,42]. Other studies have shown that neurons in these regions are bathed by circulating factors (containing LEP) and express LEP receptors, enabling a direct response to changes in LEP by altering the efferent neural outflow and secretion of endocrine factors that regulate peripheral tissues, such as bone [43]. Currently, the balance between the central and peripheral effects of LEP in bone remains elusive [43]. We may be able to explore this further through more experimental work in the future. Furthermore, previous studies have revealed that LEP is involved in a variety of biological processes by regulating the downstream signaling pathways. LEP leads to the sequential activation of p38, phosphoinositide 3-kinase (PI3K), and extracellular signal‐regulated kinase (ERK) signaling pathways to inhibit glucose absorption, increases their involvement in cell cycle regulation by activating the janus kinase 2 (JAK2)-signal transducer and activator of transcription 3 (STAT3), mitogen-activated protein kinase (MAPK)-1/3, and PI3K-serine/threonine kinase 1 (AKT1) signaling pathways, and also plays a role in cell apoptosis via the JAK2-STAT3 pathway [44–46]. Therefore, in future studies, we aim to focus on the influence of the downstream regulation mechanism of LEP on osteogenic differentiation. Moreover, we established an animal model of OP regulating miR-874-3p to further demonstrate its effect on osteogenic differentiation in OP in vivo.

## Conclusion

In conclusion, miR-874-3p is highly expressed in hBMSCs during osteogenic differentiation and is positively correlated with osteogenic gene expression. The newly discovered miR-874-3p/LEP axis provides insights about the mechanism of osteogenic differentiation of hBMSCs and may be used as a theoretical basis to develop novel strategies for the treatment of OP.

## Supplementary Material

Supplemental Material

## Data Availability

The datasets used and/or analyzed during the current study are available from the corresponding author on reasonable request.

## Highlights

1. miR-874-3p was downregulated in the femoral neck trabeculae of patients with OP, and upregulated during osteogenic differentiation.
2. miR-874-3p upregulation promoted ALP, RUNX2, OCN, and OSX mRNA expression, cell proliferation, and the deposition of hBMSCs.
3. LEP was found to be a target gene of miR-874-3p.
4. LEP upregulation inhibited the levels of osteoblast markers and reversed the effect of osteogenic differentiation induced by miR-874-3p upregulation.
