# Supplementary material for: MicroRNA miR-874-3p inhibits osteoporosis by targeting leptin (LEP)
Source: Bioengineered. 2021 Dec 11;12(2):11756–67. doi: 10.1080/21655979.2021.2009618 (PMC8810162; doi:10.1080/21655979.2021.2009618)
Supplement: Supplemental Material [file KBIE_A_2009618_SM8653.zip › supplementary/Supplementary Table 1.docx]

Supplementary Table 1. The top 5 ranked conserved target miRNAs of LEP mRNA predicted by TargetScan Human 7.2

| miRNA Name | Position in the UTR | Seed match | Context++ score | Weighted context++ score |
| --- | --- | --- | --- | --- |
| hsa-miR-9-5p | 181-188 | 8mer | -0.44 | -0.44 |
| hsa-miR-532-5p | 147-154 | 8mer | -0.3 | -0.3 |
| hsa-miR-296-5p | 1850-1857 | 8mer | -0.29 | -0.29 |
| hsa-miR-874-3p | 1897-1903 | 7mer-m8 | -0.25 | -0.25 |
| hsa-miR-668-3p | 631-637 | 7mer-m8 | -0.23 | -0.23 |
